# Supplementary material for: Minimally Invasive Preclinical Monitoring of the Peritoneal Cavity Tumor Microenvironment
Source: Cancers (Basel). 2022 Mar 31;14(7):1775. doi: 10.3390/cancers14071775 (PMC8997523; doi:10.3390/cancers14071775)
Supplement: Supplementary file 1 [file cancers-14-01775-s001.zip › cancers-1649336-supplementary.pdf]

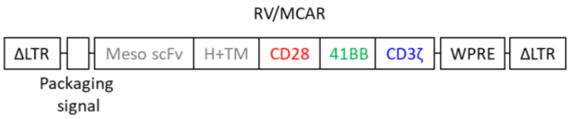

Figure S1. Schematic representation of mesothelin-targeting CAR retroviral vector**.**

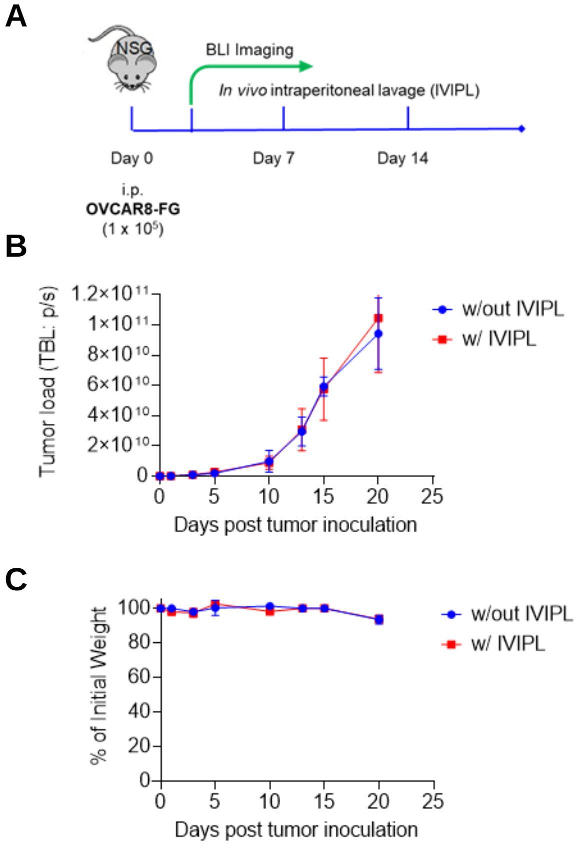

Figure S2. IVIPL does not affect OVCAR8-FG tumor growth nor mice weights. (**A**) Experimental design to monitor IVIPL affect on tumor growth and mice health in an ovarian cancer model (n=2 per group). (**B**) Tumor growth curve. (**C**) Weight measurements.

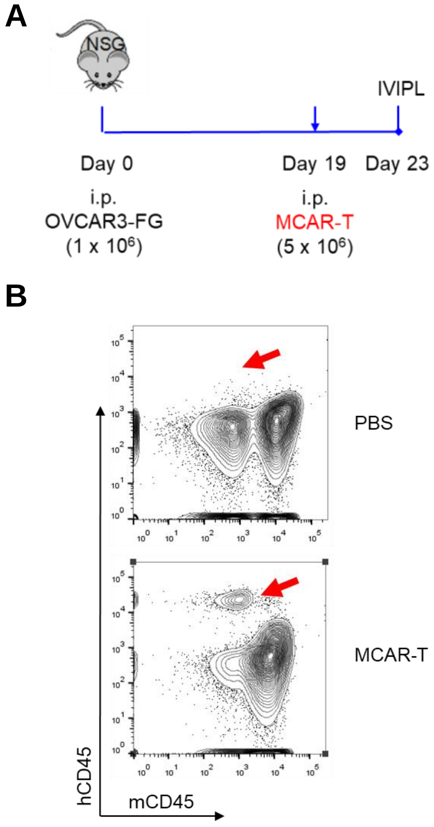

Figure S3. IVIPL identified MCAR-T cells in an OVCAR3FG model. (**A**) Experimental design to identify MCAR-T cells using IVIPL in an OVCAR3FG ovarian cancer model. (**B**) FACS plot of IVIPL fluid showing MCAR-T cells (hCD45+) in the peritoneal cavity.
